# Cilia density and flow velocity affect alignment of motile cilia from brain cells

**DOI:** 10.1242/jeb.229310

**Published:** 2020-12-29

**Authors:** Nicola Pellicciotta, Debasish Das, Jurij Kotar, Marion Faucourt, Nathalie Spassky, Eric Lauga, Pietro Cicuta

**Affiliations:** 1 Cavendish Laboratory, University of Cambridge, Cambridge CB3 0HE, UK; 2 Department of Applied Mathematics and Theoretical Physics, University of Cambridge, Cambridge CB3 0WA, UK; 3 Cilia biology and neurogenesis, Institut de biologie de l'Ecole normale superieure (IBENS), Ecole normale superieure, CNRS, INSERM, PSL Universite Paris, 75005, Paris, France

**Keywords:** Brain development, Hydrodynamic forces, Fluid flow

## Abstract

In many organs, thousands of microscopic ‘motile cilia’ beat in a coordinated fashion generating fluid flow. Physiologically, these flows are important in both development and homeostasis of ciliated tissues. Combining experiments and simulations, we studied how cilia from brain tissue align their beating direction. We subjected cilia to a broad range of shear stresses, similar to the fluid flow that cilia themselves generate, in a microfluidic setup. In contrast to previous studies, we found that cilia from mouse ependyma respond and align to these physiological shear stress at all maturation stages. Cilia align more easily earlier in maturation, and we correlated this property with the increase in multiciliated cell density during maturation. Our numerical simulations show that cilia in densely packed clusters are hydrodynamically screened from the external flow, in agreement with our experimental observation. Cilia carpets create a hydrodynamic screening that reduces the susceptibility of individual cilia to external flows.

## Abstract (precis)

**Summary:** Alignment of motile cilia in mammalian brains is essential for transport of fluids as described in an *in vitro* model of the developing brain.

## INTRODUCTION

The ventricular cavities of the brain are covered by ependymal cells bearing motile cilia (Worthington and Cathcart, 1966), which are whip-like organelles that can propagate bending waves and produce fluid flow over the cell surface. Their ability to generate fluid flow is a fascinating example of collective behaviour (Blake and Sleigh, 1974) in biology. It has been reported to have fundamental roles in microorganisms (Knight-Jones, 1954) and in many organs of mammals such as airways and fallopian tubes. In the brain, motile cilia circulate cerebrospinal fluid (CSF) thought to be necessary for brain homoeostasis, toxin washout and orientation of the migration of newborn neurons (Sawamoto et al., 2006). In mice, defects in ciliary motility are known to cause hydrocephalus (Lechtreck et al., 2008; Fliegauf et al., 2007). In zebrafish juveniles and adults, cilia-driven flow was also shown to be critical for body axis formation in the embryo and spine morphogenesis (Zhang et al., 2018).

Recent works have reported that cilia orientation is highly organised in modules in the third ventricle cavity of mice, which facilitates spatiotemporally regulated networks of fluid flows (Faubel et al., 2016). The specific function of these intricate flow patterns is still unknown. However, they may be important for the distribution of substances to targets in this ventricle, which include periventricular hypothalamic nuclei that control circadian rhythms, hormone release, thermoregulation, blood pressure, satiety and feeding (Franklin and Paxinos, 2008; Spassky and Meunier, 2017). How cilia beat reliably in a particular direction and switch periodically in the brain ventricles is currently unknown and needs further investigation. In model systems, we have some understanding of synchronisation (Bruot and Cicuta, 2016) and development of polar order (Bruot and Cicuta, 2013). A current hypothesis is that cilia beating direction in the brain is controlled by the flow present in the brain, arising from CSF production and absorption (Spassky and Meunier, 2017; Stadlbauer et al., 2010) and cilia activity itself (Guirao and Joanny, 2007).

In the brain, multiciliated ependymal cells are derived from radial glial cells during embryonic development, and their maturation is characterised by the growth of cilia from the basal bodies locked at their apical membrane (Spassky et al., 2005). In a previous study (Guirao et al., 2010), we observed that multiciliated cells in the brain are responsive to external hydrodynamic forces only during the maturation process. In that study, shear stress comparable to the one generated by cilia-driven flows *in vitro*, τ_0_≈0.02 dyne cm^−2^ was applied on brain cultures both during and after cell maturation. The value of the applied shear stress τ_0_ was chosen based on the estimated average flow, *V*, produced by a ciliated cell in culture. We measured *V*≈50 μm s^−1^ at a distance of *h*≈20 μm from the wall, leading to τ_0_≈η*V*/*h*=0.02 dyne cm^−2^, where η is the viscosity of the medium. We showed that during cell maturation, ciliated ependymal cells aligned their beating in the direction of the applied shear stress. In contrast, after cell maturation, no significant alignment was measured in cultures exposed to the same value of shear stress. These results suggest that cilia beating direction locks after complete cell maturation and cilia are no longer susceptible to flows (Guirao et al., 2010), similarly to ciliated cells in the larval skin of *Xenopus* (Mitchell et al., 2007).

However, the locking of cilia direction after cell maturation is in stark contrast with recent cilia-flow measurements in the mouse third ventricle (*in vivo*) (Faubel et al., 2016). Here, cilia-driven flow patterns were reported to reliably and periodically change, which was also the case in completely mature ependymal cells in the brain ventricles (Faubel et al., 2016). Remarkably, these authors also showed that cilia-driven flows have magnitude much higher than the one measured *in vitro* (*V*=100–500 μm s^−1^) (Faubel et al., 2016). These *in vivo* measurements highlight that the cilia-driven flow generates shear stresses of τ≈2–10τ_0_=0.04 to 0.2 dyne cm^−2^, higher than the shear stress estimated in our previous work (Guirao et al., 2010).

These recent studies motivated us to investigate the time flow response of ependymal cell culture at shear stresses similar to that experienced *in vivo*. We exposed ependymal cells to shear stress in a novel microfluidic device composed of a PDMS channel, and standard Corning Insert recently developed in our group (Pellicciotta et al., 2020). We found that cilia align with the external flow at all times in culture for shear stresses similar to the ones present in the brain. This evidence suggests that beating direction of cilia is not locked but still able to respond to flow even after cilia maturation, in agreement with recent observations *in vivo* (Faubel et al., 2016).

Moreover, we found that cilia aligned more strongly with the external flows during cell maturation than after. We suggest that this different degree of alignment with the external flow is because of multiciliated cell density increases during cell maturation. Using numerical simulations, we were able to show that hydrodynamic interactions between cilia in shear flow can be responsible for this effect. Our main conclusion is that cilia tightly packed in a cluster are shielded from the external flow. Hence, cilia density and multiciliated cell density are important parameters for the cilia response to shear flow that may have implications on the mechanism arising in the complex flow networks in the third ventricles and other organs such as the airways.

## MATERIALS AND METHODS

### Ependymal cell culture in microfluidic Transwell chips

Differentiating ependymal cells were isolated from mouse brain and grown in flasks as previously reported (Delgehyr et al., 2015). All animal studies were performed in accordance with the guidelines of the European Community and French Ministry of Agriculture and were approved by the Ethics committee Charles Darwin (C2EA-05) and ‘Direction departementale de la protection des populations de Paris’ (Approval number Ce5/2012/107; APAFiS#9343). Sex of mice was not determined.

**Fig. 1.**
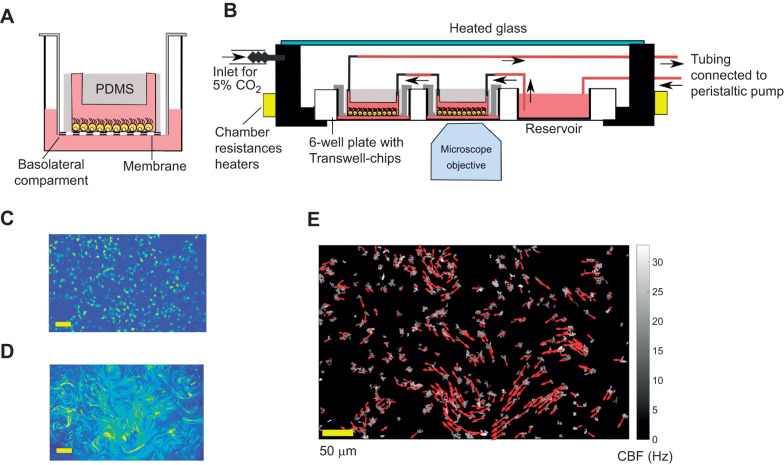
**Experimental procedure for measuring CBF and cilia beating direction in ependymal cultures treated with fluid flow.** Continuous fluid flow is applied for 3 days on cells cultured in Transwell chips. The chips are then removed from the flow and cilia motility is imaged with the microscope. From high-speed movies in bright field (BF) illumination, we identified ciliary beating frequency and cell position, while from the motion of tracer particles we inferred the ciliary beating direction. (A) Schematic of an ependymal culture in a Transwell chip. (B) Illustration of the setup: shear stress is applied on cells by cycling medium in a closed loop using a peristaltic pump and a medium reservoir. (C,D) Normalised standard deviation maps of the pixel intensity over time, from a BF movie (C) and fluorescence (FL) images after the addition of tracer particles (same field of view) (D). (E) The information from BF and FL movies merged. The colour map indicates the ciliary beating frequencies (CBF) and the red arrows the measured flow field above the identified cells. The length of the arrows is proportional to the flow velocity. The analysis procedure is also explained in Movie 1.

When cells were 70–80% confluent in T25 flasks, they were resuspended in Dulbecco's Modified Eagle's Medium (DMEM) with 10% fetal bovine serum (FBS) and 1% penicillin-streptomycin (P/S) at a concentration of 10^7^ cell ml^−1^ (Delgehyr et al., 2015). Then, cells were seeded in microfluidic Transwell chips (Pellicciotta et al., 2020). This is a novel microfluid device composed of a PDMS channel bonded to the membrane of a Corning Transwell insert (CLS3450, Sigma-Aldrich) (Pellicciotta et al., 2020). These modified Corning inserts are placed in 6-well plates, and 2 ml DMEM medium supplemented with 10% FBS and 1% P/S is also added in the basolateral compartment. The medium in the basolateral compartment acts as a nutrient reservoir that reaches the cells through the porous membrane, eliminating the need for constant media perfusion in the channel (Trieu et al., 2014; Kim et al., 2007) (see Fig. 1A). We found this extra medium to be critical for properly differentiating the neural stem cells in the channel. By contrast, PDMS channels bonded to glass were found to be inappropriate for a long term culture. The day before seeding, Transwell chips were sterilised under UV and successively coated with 320 µg ml^−1^ poly-l-lysine (Sigma-Aldrich, P1524) in Milli-Q water overnight in the incubator. Then, the devices were rinsed three times with sterile water and left to dry under the hood for 1 h.

The day after seeding (day of *in vitro* 1, DIV1), the cells in the Transwell-chip channels were first rinsed with phosphate buffered saline (PBS). Then culture medium was replaced with differentiation medium (1% P/S DMEM without FBS) in the channels and the basolateral compartments. During the following days, the medium was no longer changed. Ependymal cell maturation into multiciliated cells is achieved after approximately 10 days *in vitro* (DIV) after cell seeding (Guirao et al., 2010; Delgehyr et al., 2015).

### Peristaltic flow setup

Shear stresses of controlled magnitude were applied to ependymal cultures using a peristaltic pump setup. In contrast to the syringe pump, the peristaltic pump can be used in a re-circulating medium system. The cells produce factors that are diluted into the total culture media volume and recirculate back to the cells (Kim et al., 2007). We designed this setup because ependymal culture protocols suggest to not change the medium during differentiation (Delgehyr et al., 2015). Fig. 1B shows a schematic of the setup. The medium was pushed from the peristaltic pump to a medium reservoir using microbore tubings. In our design, the medium reservoir is inside one of the wells of the same well plate that hosts the Transwell chips. The total volume of medium in the reservoir is ∼6 ml. Then, the medium flows through the top channels of the Transwell chips, applying constant shear stress on the cells. All the Transwell chips were connected in series. The connections between reservoir and chips were made using microbore tubing and stainless steel interconnections. The medium is eventually pushed back to the peristaltic pump creating a recirculating system. The well plate (with chips and reservoir) was held in a custom-heated microscope chamber at 37°C. The correct pH and oxygenation of the cell medium and reservoir were regulated by continuously flowing air (20% O_2_) with 5% CO_2_ in the heated chamber. Two chips in the well plate were not exposed to flow and acted as a control. The flow rate of the perfused medium, and consequently the shear stress on the cell, can be tuned by changing the velocity of the peristaltic rollers.

This setup was used to expose cell cultures to shear flow for 3 days, at different stages of cells maturation. After 3 days, we removed the connections of the Transwell chips with the flow setup, and cilia motility was imaged with a microscope.

### Image acquisition procedure

Image-acquisition started 1 h after Transwell chips were disconnected from the flow setup. All videos were acquired with a microscope (Ti-E inverted Nikon, Tokyo, Japan) equipped with a 20× objective (NA 0.75). During the imaging, the well plate was held in the heated chamber with 5% CO_2_. Movie 1 illustrates the image acquisition procedure. Firstly, high-speed bright field (BF) images were acquired for several fields of view (FOVs) for each chip, usually around 20–25 FOVs per chip. Detailed statistics on the number of FOVs per chip are provided in Table S1. Each BF video was recorded at 160 frames s^−1^ for 5 s. Secondly, fresh medium with PEG-coated fluorescent particles of 1 µm diameter (particle dilution at 0.5%) (Kim et al., 2005) was added in the top channel of the Transwell-chips. The addition of this medium was done using a syringe pump previously connected to the inlet of the Transwell-chip. Each chip was held stable in the well plate using sterile tape to avoid drifting of the chip during particle addition. After 15 min, videos of the cilia-propelled fluorescent particles (FL) were recorded with fluorescent microscopy (20× objective, NA 0.75) on the previous FOVs (10 s at 10 frames per seconds) (Fig. 1D).

In the rest of the article, we indicate with ‘BF’ the bright field videos acquired before the addition of the particles. Moreover, we use ‘FL’ when referring to the videos of the fluorescent particles driven by the cilia flow. It is worth noting that ciliary beating frequency (CBF) and cell positions could not be extracted from the FL videos because particles moved around the field of view, obstructing cilia motion. For this reason, avoiding drift during particle perfusion is a critical step to obtain a precise mapping of CBF and cilia beating direction for each cell.

### Image analysis

Image acquisition and analysis are summarised in Movie 1. For each field of view, we have a bright field video of the cilia motility (BF) and a video of the FL. Each video is analysed with custom MATLAB scripts to obtain the following information. (1) Cell position: using the BF video, cell locations were first roughly identified by the pixels for which the standard deviation of the intensity over time was larger than a threshold value (found with Otsu's method) (Fig. 1C). For each of these validated pixels, CBF was then calculated as the frequency with the highest peak in the Fourier Transform signal of pixel intensities over time (Sisson et al., 2003; Dimova et al., 2005; Ryser et al., 2007). Single multiciliated cells were identified as the group of geometrically connected pixels where the frequency standard deviations did not vary more than a threshold 
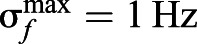
. (2) Ciliary beating direction: we assumed, as recently validated in Faubel et al. (2016) that the cilia-driven flow corresponds to the beating orientation of underlying cilia. The fluid flow generated by the ciliated cells was measured from the FL video with particle image velocimetry (PIV) method PIVLab, MATLAB (Thielicke and Stamhuis, 2014). Using a PIV algorithm, we extract a displacement vector field between all consecutive frames of the movie. Then, the displacement vector fields were averaged over time to obtain a smooth result of the flow field. For the PIV measurements, we used a grid with a spacing of 32 pixels (7.6 µm). In this way we can think of each field of view as divided in boxes of area *A*_box_=7.6×7.6 µm^2^, where for each box we have the corresponding displacement vector. The box size is roughly the size of a multiciliated cell, so we assumed that each displacement vector corresponds to an individual multiciliated cell only. We kept only the boxes which contained identified ciliated cells.

Eventually, for each selected box (or cell) *i*, we have: (1) the CBF, using the average CBF inside the box; (ii) the beating direction 
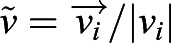
, where *v*_*i*_ is the measured flow field from PIV. Overall, combining the image analysis of videos of cilia motility and tracer particles, we can routinely measure ciliated cell positions, beating frequency and direction over all the investigated FOVs (Fig. 1E). For each field of view, we usually identify between 100 and 400 cells. Image acquisition and analysis steps are summarised in Movie 1.

## RESULTS

### Alignment of cilia beating with shear stress depends on the maturation stage

**Fig. 2.**
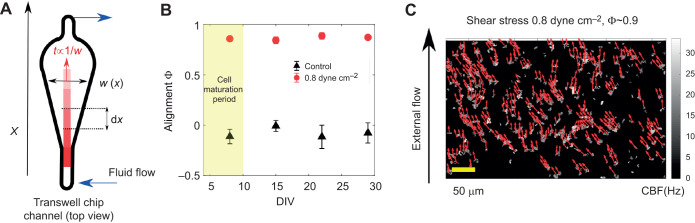
**Shear stresses from external flow align with motile cilia beating direction.** (A) Illustration of the tapered channel used to apply different shear stresses in the same cultures. The shear stress τ decreases along the main axis of the channel (*x*) as the channel width *w* increases. Movies were recorded only along the centre line of the tapered channel. For each tapered channel (Transwell chip), we acquired 20–25 fields of view (FOVs). Since the shear stress changes continuously along the main axis of the channel, the alignment parameter and the shear stress has been averaged over segments of length δ*x*=1700 μm. With our equipment, we have five fields of view for each segment. Each field of view contains 100–400 identified cells. (B) Ciliary beating strongly aligns with the external flow for τ≈0.8 dyne cm^−2^. We found no difference in the alignment for cell treated with shear stress during or after cilia maturation (maturation period marked in yellow; days *in vitro* DIV≤10). Each point is the alignment parameter Φ averaged over at least 20 fields of view (more information on the statistics is provided in Table S1). Error bars are the standard error of this average. (C) Analysis results for a field of view in a culture at 29 DIV and treated with τ≈0.8 dyne cm^−2^. The bright field video of cilia motion and propelled particles are available in Movie 2.

Culture of cells was carried out in Transwell chips with tapered channels whilst a shear stress was applied. The tapered geometry of the microfluidic channel in the Transwell chip allowed us to have different strengths of shear stress applied onto the same culture, whilst maintaining a constant flow rate (Rossi et al., 2009). The width of the channel increases as a function of the axial position *x*, and this leads to a hyperbolic shear stress along *x*, τ=6η*Q*/(*h*^2^*w*) with *w*(*x*)=*w*_*x*=0_+α*x*, Fig. 2C. In our design, the height is *h*=1 mm, while the width spans from *w*_0_=1 mm to *w*_max_=5 mm, with a slope of α=15 deg. The length of the tapered section is *l*=7 mm. With this design, in the same chip, we can apply shear stresses that span across over half an order of magnitude, τ(*x*=0)≃5τ(*x*=*l*). More information on the flow rates used is provided in Table S1. In Fig. S1, we show measurements of the shear stress in the channel, as a function of the position in the tapered channel, for the flow rates used in the experiments.

We applied a wide range of shear stresses τ∈[0.02, 0.8] dyne cm^−2^ on cultures of ependymal cells at different time points of their biological maturation process, from radial glial cells to multiciliated ependymal cells. In agreement with past studies (Spassky et al., 2005; Guirao et al., 2010), ependymal cells are considered mature after 10 days *in vitro* (DIV). The shear stress is maintained constant over 3 days. We applied shear stress on cultures starting from from DIV 5, 12, 19, 26 up to respectively DIV 8, 15, 21, 29. Each shear stress treatment has been repeated for at least 3 Transwell chips, see also Table S1. The range of shear stress was chosen in agreement with the shear stress shown to be generated by the cilia-driven flow in the brain (Faubel et al., 2016).

After the 3 days of constant fluid perfusion, the Transwell chips were disassembled from the flow-setup and cells imaged with and without tracer particles, following protocols described in the Materials and Methods. For numerous cells in the centre of the channels, we gathered data of their position, ciliary beating frequency (CBF), magnitude and direction of the cilia driven flow 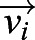, where *i* is the index corresponding to the box containing the cell. These measurements were performed in the absence of external flows. We measured the global cilia alignment using an alignment parameter defined as:(1)
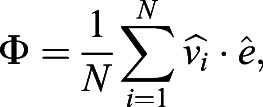
where 
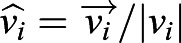
 is the unit vector pointing in the direction of the cilia-driven flow, 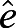 is the unit vector pointing in the direction of the previously applied external flow, and *N* is the total number of boxes where ciliated cells are detected. During these measurements, there is no external flow applied and the fluid flow 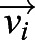 is only cilia-driven. The values Φ=1 and Φ=0, correspond to cilia beating directions that are either completely aligned or randomly oriented with the applied flow, respectively.

We found that the cultures, once treated with shear stresses of strengths similar to what is found in the brain, align their beating direction almost completely with the external flow. Remarkably, this is independent of the cell maturation stage. This is evident for cells treated with the highest shear stress, *τ*≈0.8 dyne cm^−2^ (see Fig. 2A): the measured global alignment is Φ≈0.9, independently of cilia maturation. These cultures were able to generate a net average flow in the direction of alignment. We measured this net flow as:(2)
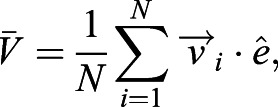
and found 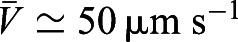 for mature cells, while 
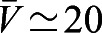
 μm s^−1^ for cells treated during the maturation stage (Fig. S2). By contrast, we did not find any significant global alignment in control cells that were not exposed to shear stress, in agreement with previous studies (Guirao et al., 2010). In the control cultures, cilia had a short-scale alignment (see also Fig. 1E) but the average global alignment was approximatively zero, i.e. Φ≃0, as expected for cilia beating in random directions (Fig. S4).

**Fig. 3.**
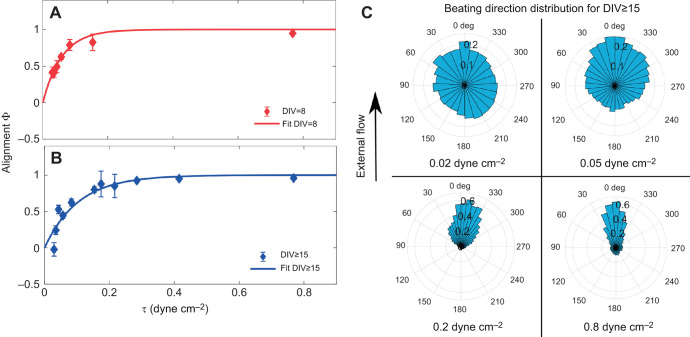
**Cilia beating alignment refines with the shear stress applied, both during and after cell maturation.** Data represents the alignment parameter Φ as a function of the applied shear stress Θθτ∈[0.02, 0.8] dyne cm^−2^ for (A) cultures treated with flow during cell maturation (DIV5–8) and (B) cultures treated after maturation (cells imaged at DIV≥15). Both datasets in A and B are fitted with the empirical function 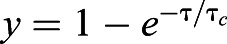. Each data point is the alignment parameter Φ averaged over at least 20 fields of view (more information on the statistics in Table S1). Error bars are s.e.m. Each field of view contains between 100 and 400 cells. Cultures grown in tapered channels but not treated with shear stress do not show a global alignment, Fig. S4. (C) Distribution of cell beating directions treated with shear stress after cell maturation (data for cultures treated after maturation, and imaged at DIV≥15) as a function of the applied shear stress. Each graph has been extracted from at least 20 fields of view.

Cilia alignment does depend on cell maturation: this is clear by applying the lowest range of shear stresses. For shear stress τ=0.03 dyne cm^−2^ we measured the alignment parameter Φ to be positive and significantly different from zero, Φ=0.5, for cells treated during maturation. In contrast, the alignment parameter is approximately zero for cells with same flow treatment but at a mature stage, see Fig. 3. These measurements are in agreement with the results previously obtained (Guirao et al., 2010), where the alignment of the ciliary beat with shear stress τ=0.018 dyne cm^−2^ was only observed for cultures treated during cell maturation (DIV≤10).

It is worth mentioning that, similarly to the *Xenopus* ciliated skin (Mitchell et al., 2007), no significant alignment was observed with mature cells at DIV 15, after treatment with high oscillatory shear stress (τ_osc_=0.3 dyne cm^−2^ during the half-cycle), as shown in Fig. S3.

In Fig. 3A,B we show the alignment parameter Φ as a function of the applied shear stress, for the cultures treated before and after maturation. In both cases, we found that ciliary beating alignment increases with the applied shear stress. We fit these curves with an empirical function y=1–exp(–τ/τ_c_). This function agrees reasonably well with most data points, and can be used to extract a parameter τ_c_ characterising the response of the cell culture at different maturation stages. We can think of τ_c_ as a critical value for the shear stress needed to induce a significant cilia alignment, the threshold Φ(τ_c_)≈0.6. For cultures treated at a mature stage, we found τ_c_=0.1±0.05 dyne cm^−2^, approximatively twice that obtained for cell treated during maturation, τ_c_ =0.05±0.02 dyne cm^−2^. In both cases, the shear stress generated by cilia-driven flow measured in the brain *in vivo*, (τ≈0.04–0.2 dyne cm^−2^; Faubel et al., 2016) would be enough to have a strong influence on the beating direction of neighbouring cilia.

A reduction of cilia susceptibility could be caused by the increased density of ciliated cells during culture maturation. For example, this can be due to a rise in the number of multiciliated cells. Indeed, during the maturation of ependymal cells, the percentage of ciliated cells increases significantly (Guirao et al., 2010). In turn, an increase in the density of cilia in the epithelium promotes more hydrodynamic screening of the external flow, as less fluid can creep past a dense carpet of cilia. In Fig. S8, we show the trend of the alignment Φ as a function of the density of multiciliated cells during the maturation period. In our devices, we measured the density of multiciliated cells, ρ_cell_ during and after differentiation by measuring the total number of boxes where ciliated cells are identified (see Materials and Methods), for each field of view. The ciliated cell density is then found as ρ_cell_=*N*_box_/(*N*_tot_×*A*_box_), where *N*_box_ is the number of boxes with ciliated cells, *N*_tot_ is the total number of boxes in a field of view and *A*_box_ is the box area. In agreement with our previous studies (Guirao et al., 2010), we found that the ciliated cell density almost doubled comparing halfway in cell maturation to after. We measured a ciliated cell density of ρ_cell_=1500±100 cells mm^−2^ for cells at DIV=8 and ρ_cell_=2900±100 cells mm^−2^ for differentiated cells at DIV≥15. Each value is an average over eight cultures.

In order to estimate the hydrodynamic screening effect of dense cilia, it would be interesting to directly measure cilia density in our cultures. However, with our setup, we did not have the resolution to directly measure the density of cilia and beating direction at the same time in the epithelium. Alternatively, we can account for the change of cilia density during maturation by measuring the variation of the fraction of area cover by cilia while beating. Assuming that all the boxes where we identified ciliated cells are completely covered by the movement of beating cilia (see Materials and Methods), we can define the fraction of area cover by beating cilia as ρ=*N*_box_/*N*_tot_. From this parameter, knowing the area spanned by each individual cilium during its beating would give us the cilia density. It is worth noticing that an exact calculation of the cilia density from the area fraction would also require to know the overlapping area between cilia within the same cell. We found ρ=0.09±0.01 for cells at DIV=8 and ρ=0.17±0.01 for differentiated cells at DIV≥15 (see also Fig. S5). In the next section, we numerically estimate the trend of this hydrodynamic screening effect as a function of ciliated fraction area and cilia density.

### Hydrodynamic screening in cilia arrays: numerical simulations

Using accurate hydrodynamic simulations, we demonstrate that the effect of an externally imposed fluid flow on a closely packed collection of ciliary filaments is reduced because of hydrodynamic screening (or shielding). In other words, the hydrodynamic drag forces due to the imposed flow reduce in strength when the density of cilia increases. We show that this screening effect has a significant impact on cilia alignment with an imposed shear flow.

The fluid dynamics of beating cilia, owing to their relatively small size (≃10  μm length and 
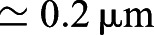
 width), are described by the incompressible Stokes equations (Lauga and Powers, 2009). The hydrodynamics of an arbitrarily shaped body using the Stokes equation is best solved numerically using the boundary element method (Pozrikidis, 1992). However, if the body under consideration is slender, then the boundary element method requiring surface integrations reduces to a contour integral called slender body theory (Johnson, 1980; Das and Lauga, 2018, 2019). Here, we employ slender body theory to study the hydrodynamics of multiple filaments exposed to shear flow. A detailed description of the simulation methods can be found at https://doi.org/10.5281/zenodo.4305524.

**Fig. 4.**
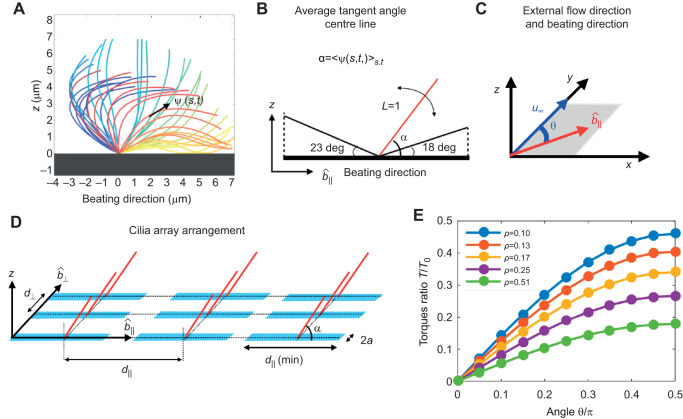
**Numerical calculations of hydrodynamic torque on cilia arrays** **of varying density.** (A) The beat pattern of a single cilium from ependymal culture, data taken from Hamilton et al. (2020), is taken as an input. The cilium centre line is characterised by its tangent profile ψ(*s*,*t*) as a function of the arclength along the cilium *s* and at the time *t*. (B) We model each cilium by a rigid rod attached to a surface, tilted to an angle, calibrated from the cilium beat pattern. In the figure we also mark the angle that the cilium reaches during the transient period between power and recovery stroke. (C) An external shear flow *u*_∞_ is applied along the *y*-axis; cilia beating direction is identified with the unit vector 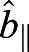, in the *x–y* plane. The angle between the cilia beating direction and the external flow is θ. (D) Cilia carpets are then represented by regular arrays of *N*_*r*_ cilia, all tilted with an angle α along the beating direction 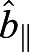. (E) The average hydrodynamic torque on an individual cilium, as a function of the angle with the external flow, θ, and cilia density ρ. The torque experienced by an individual cilium in a dense array of cilia in a shear flow decreases as the cilia density increases. The reported torques are normalised with *T*_0_(θ=π/2)=0.55, which is the torque experienced by an isolated single cilium.

The beating pattern of mammalian cilia is generally more extended in the direction of the power stroke (Hamilton et al., 2020). For this reason, in the presence of a constant external flow, a beating cilium feels a net average torque during its beating cycle (Guirao and Joanny, 2007). To quantify this effect, we first analyse the beating pattern (waveform on the cilium, over time) of brain cilia. The position of a cilium centreline during the beating cycle is available in the literature for mature ependymal cells (Hamilton et al., 2020). We characterised the cilium centreline by its tangent angle profile ψ (*s*,*t*) as a function of the arclength along the cilium, *s*, and time, *t* (see Fig. 4A). We next measured the average tangent along the cilium arclength during one beat period. We found this vector to be at an angle α=53 deg with respect to the direction of the power stroke. Therefore, in multiciliated ependymal cells, the beating pattern is slightly more extended in the direction of the power stroke.

In our simulation, the cilia carpet is represented by an array of *N_r_* static rods protruding from the *x–y* plane (in the *z* direction). The length of each rod is normalised to *L*=1 and radius is *r*=0.02 (see Fig. 4B). In order to coarse-grain the asymmetry of the beating pattern, all these rods are tilted at an angle of α=53 deg with respect to the beating direction (Fig. 4C). An estimation of the error made with this assumption is reported in Fig. S7. In our model, all the cilia have the same beating direction, which we identify with the unit vector 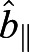, lying in the *x–y* plane. The distance between individual cilia is such that the beating patterns do not overlap with each other at any given point in time. The minimum distance between two cilia perpendicular to the beating direction, *d*_⊥_ is assumed to be two body widths in length, *d*_⊥_(min)=0.08. On the other hand, along the beating direction, the minimum distance is 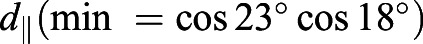, where 23 deg and 18 deg are the maximum tilt angles with respect to the surface reached by the cilium (see Fig. 4B).

In these simulations, cilia carpet is represented by an ordered array of cilia; thus we are not including the fact that in multiciliated cell cultures, cilia are arranged in heterogeneous patches (multiciliated cells). However, we express cilia density in terms of ciliated fraction area. In this way, a comparison between simulation and experiment is possible. In the experiments, we estimate the density of cilia with the ciliated fraction area, ratio of the cilia beating spanned area, viewed from the top, to the entire field of view. Consistent with the experiments, here we defined cilia density by using the cilia-spanned area of one cilium when viewed from the top. From the previous analysis of the beating pattern, we know this area to be 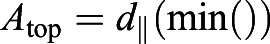. Then the ciliated fraction area in our simulations, ρ, is simply calculated as the fraction of area occupied by the cilia, *N*_*r*_*A*_top_, and the simulation area (see Fig. 4D). It is worth noting that in the simulations cilia spanned areas never overlap, whereas these do overlap in the same multiciliated cells. Then an exact comparison between the two would require knowledge of the cilia spanned area overlap. We then changed the ciliated fraction area in the simulations by changing the distance between cilia in the direction perpendicular to the beating (*d*_⊥_=0.08, 0.16, 0.24, 0.32, 0.40). The shear flow acts as a forcing for the system that is solved numerically. The calculation method of the net force and torque on each filament is provided at https://doi.org/10.5281/zenodo.4110844. Since the cilia are very close to each other in the beating direction, the strongest hydrodynamic interactions of a cilium is with its neighbour in the perpendicular direction. The results presented here are for the smallest spacing in beating direction, 
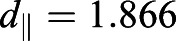
. On increasing the spacing 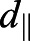, we did not find any discernible changes in the hydrodynamic screening effect. Lastly, the effect of a rigid surface is modelled using appropriate images that satisfy the no-slip hydrodynamic boundary condition by construction. The surface is placed at a small distance of 0.05 below the cilium's immobile end, to avoid numerical singularities in the slender body theory method, as described at https://doi.org/10.5281/zenodo.4305524.

We impose a shear flow, 
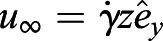
, along the *y*-axis, where 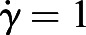 is the dimensionless shear rate. The angle between the cilia beating direction, 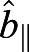, and the shear flow is denoted as θ (Fig. 4C). The no-slip velocity boundary condition applies on the substrate. When θ=0, the external shear flow is in the same direction as that of the beating cilia, and does not produce any hydrodynamic torque. On the other hand, when θ=π/2, the flow is perpendicular to the beating filaments and produces the maximum hydrodynamic torque.

We first measured the torque along the *z*-direction on an isolated filament in shear flow. When θ=π/2, the isolated filament experiences a torque of *T*_0_(θ=π/2)=0.55, non-dimensionalised by 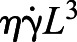. Then, we compare this values with the average torque acting on a filament in dense arrays, *T*(θ,ρ), as a function of the angle θ and for various cilia densities ρ. In Fig. 4E we report these torques, *T*(θ,ρ), normalised by the torque on the isolated filament *T*_0_(θ=π/2). We found that the torque experienced by a filament in a dense array significantly decreases with cilia density. The trends shown in Fig. 4E demonstrate the strong hydrodynamic screening effect. We then compare the torques for a ciliated area fraction similar to the one measured in the ependymal maturation period (ρ≈0.1) and after maturation (ρ≈0.2). For an angle of θ=π/2 and density ρ=0.1, the torque on the cilia almost halves, specifically *T*_0_(π/2, ρ≈0.1 ). Therefore, the weaker alignment of cilia after the cell maturation period could be simply a consequence of hydrodynamic screening.

**Fig. 5.**
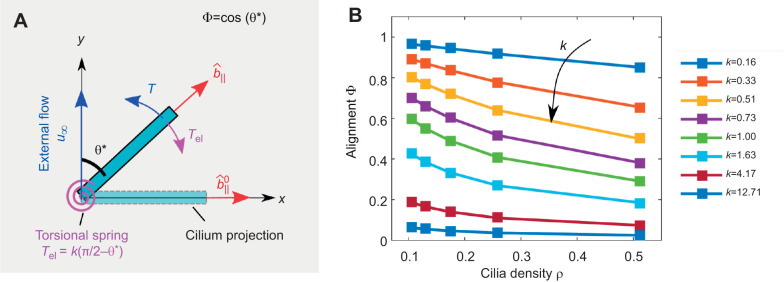
**Hydrodynamic torque on cilia arrays.** (A) We modeled the cilia alignment by allowing the cilia beating direction to rotate around the *z*-axis in response to the hydrodynamic torque. In the absence of external flow, the beating direction 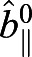 is along the *x*-axis. Then, the external shear flow is fixed along the *y*-direction. The cilia beating direction, 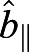, is identified with θ, the angle between the beating direction and the external flow. The hydrodynamic torque acting on each cilium (*T* in blue) is resisted by an elastic torque acting at the base modelled as a linear torsional spring (*T*_el_ in purple). The balance between the two torques leads the cilium to an equilibrium angle, θ*. In this geometry, the alignment parameter of the cilium with the external flow can be calculated as 
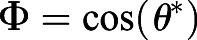
. The illustration represents only one cilium in an dense array of cilia. (B) The alignment parameter Φ for various cilia density ρ and spring stiffness *k*. Depending on the spring constant, densely packed cilia undergo lesser alignment as their neighbours hydrodynamically screen them. The spring stiffness reported here is the non-dimensional value, and can be related back to a physical system by the factor 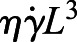, where η is the viscosity, 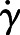 the shear rate and *L* the length of the filament.

Next, we modelled the cilia alignment phenomenon by allowing the beating direction 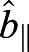 of the slender filaments to change. The schematic of the problem is illustrated in Fig. 5A. In the absence of external flow, the beating direction 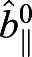 is along the *x*-axis. Then, we impose an external shear flow along the *y*-direction so that θ=π/2. The hydrodynamic torque caused by the shear flow is resisted by an effective elastic torque acting at the base of the ciliary array, which represents the response of the actin mesh to which the cilia are anchored (Mahuzier et al., 2017). This elastic torque is modelled as a torsional spring that is a linear function of θ:(3)
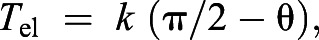
where *k* is the effective spring's stiffness. Cilia beating direction reaches an equilibrium angle denoted as θ* when the hydrodynamic torque acting on the cilium balances the elastic torque arising from the base (Fig. 5A). The equilibrium angle θ* is simply obtained by the intersection of the hydrodynamic and elastic torques, see Fig. S6. The equilibrium angle θ* leads to the alignment parameter 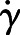. The alignment parameter, Φ, is plotted as a function of various ciliated area fractions ρ and torsional spring stiffness in Fig. 5.

### Discussion and conclusions

Our results provide new valuable insights into the mechanism of cilia alignment in the brain. Contrary to past findings (Guirao et al., 2010), our measurements show that cilia can respond and align to physiological shear stress found in the brain even after complete cell maturation. Specifically, we observed that the shear stress needed to align mature cells significantly is around τ≃0.1 dyne cm^−2^. Our results are particularly important because of recent observations in mouse ventricles of switching of cilia-driven flow patterns (Faubel et al., 2016); indeed, those observations of cilia direction switches in the brain could be then caused by time dependence of cilia activity or of CSF production.

We have also measured that the shear stress needed to significantly align cilia during the cell maturation period is around τ≈0.05 dyne cm^−2^, i.e. two times weaker than what is required for mature cells. Here, we suggest that this phenomenon is caused by the increase of ciliated cell density during culture maturation. Indeed, during the maturation period, the percentage of area covered by ciliated cells increases significantly as shown previously (Guirao et al., 2010). In turn, higher cilia density promotes higher hydrodynamic screening of the external flow (Pellicciotta et al., 2020), because less fluid can creep through a dense carpet of cilia. This hypothesis was demonstrated here in silico: our simulations show that densely packed cilia feel a lower average torque owing to the external flow, when their neighbours hydrodynamically screen them. As a result, the alignment of cilia with external flow decreases as the density of the cilia carpet increases. Using the measured values of the ciliated area fraction during and after cell maturation, we found that the hydrodynamic torque on the single cilium is expected to halve after maturation. Thus, hydrodynamic screening could be sufficient to explain the decreased cilia alignment with the flow after maturation. We modelled the resistance of cilia to align with the external flow as arising at the attachment point on the surface, with a simple linear torsional string.

We cannot exclude the possibility that other mechanisms are at play in the cilia flow susceptibility during cell maturation. For example, during multiciliated cell maturation, an actin network is established between cilia basal bodies (Kulkarni et al., 2018) and can resist the shear stress caused by the cilia beating (Mahuzier et al., 2017). This three-dimensional actin structure embeds cilia from their distal appendages to their rootlets. In principle, the increase of this actin network could also be responsible for the lower susceptibility of cilia beating to shear flow during maturation. Further experiments are needed to accurately characterise the elasticity (and viscosity) of this apical actin network during cell maturation. Possible approaches could include the manipulation of the cilium through optical or magnetic tweezers. Nevertheless, our simulations clearly highlight that hydrodynamic screening is a strong effect which changes with cilia density, and it must be taken into account when studying cilia alignment in external shear flow in any organism. Previous work concluded that cilia alignment is possible only during differentiation without taking into account this critical effect (Guirao et al., 2010; Mitchell et al., 2007). Hydrodynamic screening and cilia density in the brain may play an essential role in how CSF establishes these complex flow networks during development (Guirao et al., 2010). During *in vivo* brain development, when ciliated cells acquire rotational polarity, all the basal bodies in a single cell migrate simultaneously in the anterior of the cell (Hirota et al., 2010). This process, called translational polarity, significantly increases the local density of cilia within a cell. In light of our results, it may be that the biological significance of this densification is to reduce the cilia's susceptibility to external flows, whilst also increasing the force coupling through flow within the cilia patch. Cilia density varies greatly between mammalian organs (Spassky and Meunier, 2017), thus we could expect differences in cilia response to fluid flow depending on the organ. For example, in the airways, multiciliated cells carry up to 300 cilia per cell (Knight and Holgate, 2003), so cilia density can be almost ten-fold higher than that in the brain. Therefore, we expect hydrodynamic screening effects to be particularly relevant in this organ, making cilia alignment less dependent on external flows. In agreement with this observation, one recent study reported that cilia in airway cultures do not align with the physiological shear flow (Trieu et al., 2014), and the role of hydrodynamic forces in cilia alignment in this organ is still under debate (Vladar et al., 2012; Matsuo et al., 2013). We suggest that cilia hydrodynamic screening should be taken into account when studying the establishment of cilia beating direction in any ciliated organ.
